# *Wolbachia* infection in native populations of *Blattella germanica* and *Periplaneta americana*

**DOI:** 10.1371/journal.pone.0284704

**Published:** 2023-04-20

**Authors:** Nayyereh Choubdar, Fateh Karimian, Mona Koosha, Jalil Nejati, Razieh Shabani Kordshouli, Amrollah Azarm, Mohammad Ali Oshaghi

**Affiliations:** 1 Department of Medical Entomology and Vector Control, School of Public Health, Tehran University of Medical Sciences, Tehran, Iran; 2 Department of Parasitology, Pasteur Institute of Iran, Tehran, Iran; 3 Health Promotion Research Center, Zahedan University of Medical Sciences, Zahedan, Iran; 4 Department of Medical Entomology and Vector Control, Health Sciences Research Center, School of Public Health, Mazandaran University of Medical Sciences, Sari, Iran; University of South Dakota Sanford School of Medicine, UNITED STATES

## Abstract

Cockroaches are significant pests worldwide, being important in medical, veterinary, and public health fields. Control of cockroaches is difficult because they have robust reproductive ability and high adaptability and are resistant to many insecticides. *Wolbachia* is an endosymbiont bacterium that infects the reproductive organs of approximately 70% of insect species and has become a promising biological agent for controlling insect pests. However, limited data on the presence or strain typing of *Wolbachia* in cockroaches are available. PCR amplification and sequencing of the *wsp* and *gltA* genes were used to study the presence, prevalence and molecular typing of *Wolbachia* in two main cockroach species, *Blattella germanica* (German cockroach) and *Periplaneta americana* (American cockroach), from different geographical locations of Iran. The *Wolbachia* endosymbiont was found only in 20.6% of German cockroaches while it was absent in American cockroach samples. Blast search and phylogenetic analysis revealed that the *Wolbachia* strain found in the German cockroach belongs to *Wolbachia* supergroup F. Further studies should investigate the symbiotic role of *Wolbachia* in cockroaches and determine whether lack of *Wolbachia* infection may increase this insect’s ability to tolerate or acquire various pathogens. Results of our study provide a foundation for continued work on interactions between cockroaches, bacterial endosymbionts, and pathogens.

## Introduction

Cockroaches have survived on Earth for more than 300 million years, virtually unchanged. They are one of the most successful groups of animals because of their adaptability to various environmental conditions [1]. There are about 4500 species of cockroaches in the world, among which about 30 species frequently cohabit with human populations, and a few are indoor pests that are important in medical, veterinary, and public health fields [2, 3]. The indoor health pests prefer humid, dark, and dirty environments, where they are exposed to various microorganisms [4–6].

As an ideal biological vector, the cockroaches can acquire and mechanically transmit diverse human pathogens, including bacteria, fungi, and parasites, thereby causing serious human diseases [4, 7–10]. German cockroaches *Blattella germanica* Linnaeus 1767, (Blattodea: Blattidae) and American cockroaches *Periplaneta americana* Linnaeus 1758, (Blattodea: Blattidae) are, respectively, the first and second most contaminated cockroach species that threaten human health [11]. At the same time, cockroaches are an important source of allergens, and their faeces, debris, and secretions may cause severe allergic reactions, such as allergic asthma [12–14]. All cockroaches, but particularly the German and American cockroaches, which live in close contact with human, are also important reservoirs and transmission vectors of antibiotic resistance bacteria and/or antibiotic resistance genes [15–17].

In recent years, many studies have shown that cockroaches are rich in microbes [3, 4, 16, 18], and some of these microbes have formed an interdependent symbiotic relationship with the host during the long-term co-evolution process. *Wolbachia* is a gram-negative, maternally inherited, intracellular Rickettsia-like alphaproteobacterium that is found in many arthropods, such as insects, mites, ticks, arachnids, terrestrial crustaceans (Isopods) and filarial nematodes [19]. This bacterium is one of the most successful organisms on earth and is found globally, infecting from 20–76% of all insects [20, 21]. The bacterium acts as a reproductive parasite in arthropods where it manipulates its host’s reproduction capacity, causing a variety of abnormalities including cytoplasmic incompatibility (CI), feminization of genetic males (FM), male killing (MK) and thelytokous parthenogenesis (TP) [22–24].

It is known that bacterial endosymbionts may provide their host with nutritional benefit or with protection against xenobiotics, parasites and infections, insecticides, natural enemies, and abiotic stresses [25]. For example, *Wolbachia* produces B vitamins to support bed bugs [26, 27], or could support fly development and buffers against nutritional stress resulted in reduced pupal mortality, increased adult emergence, and larger size in several fly genotypes [28, 29]. It is suggested that *Wolbachia* acts as a nutritional symbiont to supplement insect development and increase host fitness: a selective advantage that could promote to its high occurrence in nature [28]. Certain endosymbionts may also alter acquisition and transmission of pathogens particularly arboviruses by insect vectors [30]. *Wolbachia* has been testified to suppress a variety of pathogen infections in *Aedes*, *Anopheles*, or *Culex* mosquitoes by either competition for limited nutrients or induced host immune responses [31–37].

Although *Wolbachia* is widely reported in many groups of insects, but so far limited studies on *Wolbachia* in cockroaches have been carried out. *Wolbachia* infections have been reported in a few cockroaches such as *Blattella sp*. and *Supella longipalpa* [38]. The present study was carried out to detect the presence, infection rate, distribution, and phylogeny of naturally acquired *Wolbachia* infection in cockroach populations of *B*. *germanica* and *P*. *americana* in Iran.

## Materials and methods

### Ethics statement

The protocols were conducted in this study followed the guidelines of the institutional ethical committee (Tehran University of Medical Sciences, TUMS). The protocols were approved by TUMS ethical committee under registry IR.TUMS.SPH.REC.1400.112.

### Specimen collection and morphological identification

**Fig 1:**
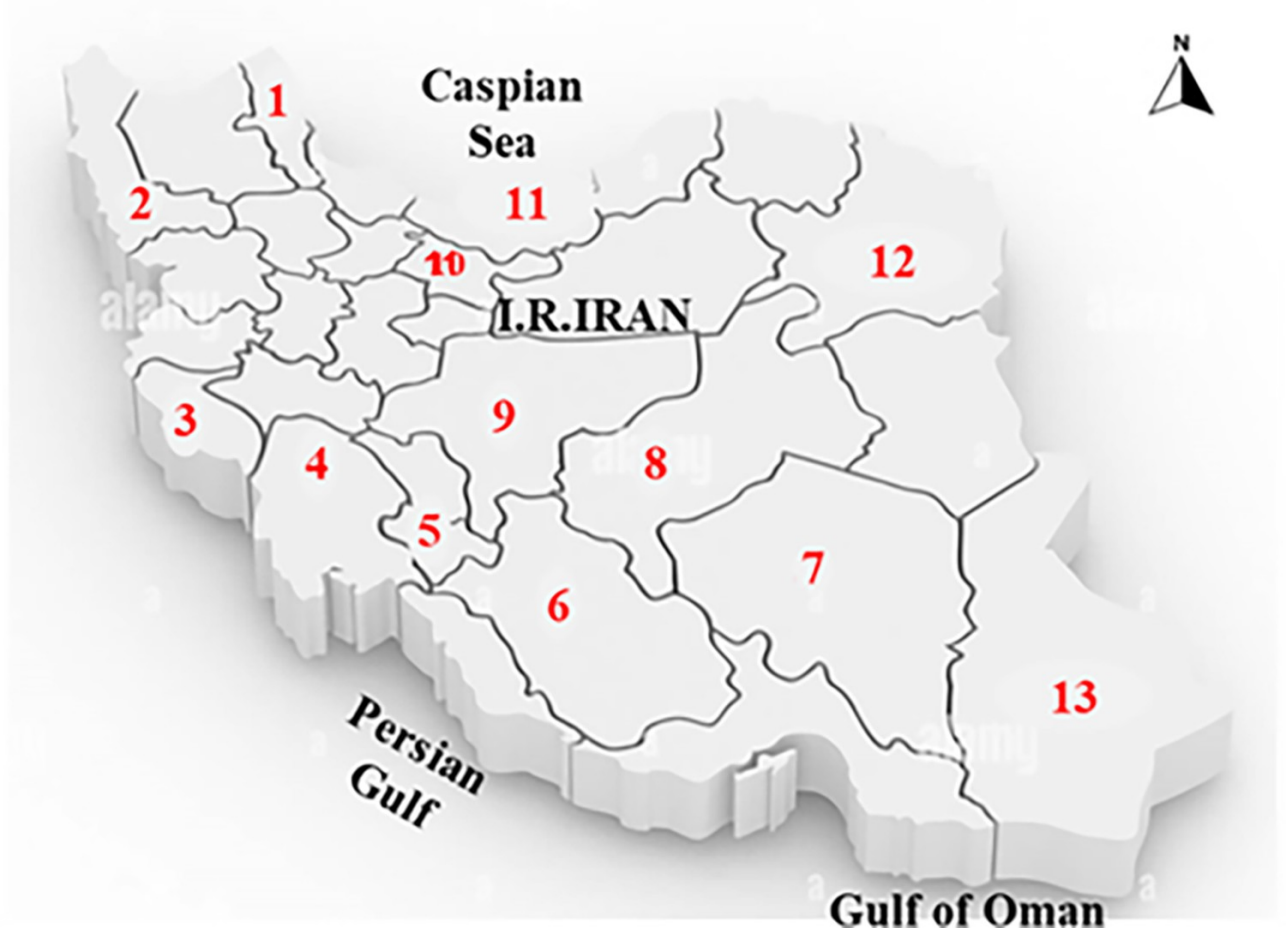
Map of sampling sites for *B*. *germanica* and *P*. *americana* across Iran. Numbers are 1: Ardabil, 2: West Azarbaijan, 3: Ilam, 4: Khuzestan, 5: Kohgiluye and Boyer-Ahmad, 6: Fars, 7: Kerman, 8: Yazd, 9: Isfahan, 10: Tehran, 11: Mazandaran, 12: Razavi Khorasan, 13: Sistan and Baluchestan. Reprinted from Choubdar et al 2021 under a CC BY license, with permission from PLOS publisher, original copyright. (https://commons.wikimedia.org/wiki/File:Map_of_Iran.png).

Cockroach specimens were collected from 13 sampling sites across the country between April and November 2021 (Fig 1, Table 1).

**Table 1:** Details of cockroach specimens collected in this study.

Province (City)	*B*. *germanica*				*P*. *americana*				Total
	Adult		Immature		Adult		Immature		
	F	M	N	E	F	M	N	E	
Ardabil (Meshghinshahr)	9	12	0	0	7	5	2	0	35
Fars (Shiraz)	32	26	7	0	12	8	0	0	85
Ilam (Ilam)	0	0	0	0	41	15	5	0	61
Isfahan (Isfahan)	25	19	0	3	11	8	2	0	68
Kerman (Kerman)	26	20	2	0	16	11	4	0	79
Khorasan Razavi (Mashahad)	18	21	0	0	22	20	0	0	81
Khuzestan (Dezful)	26	19	4	0	15	17	1	0	82
Kohgiluyeh & Boyer-Ahmad (Yasuj)	25	22	0	3	8	4	0	0	62
Mazanderan(Sari)	21	24	0	0	14	18	0	0	77
Sistan & Baluchistan (Zahedan)	27	12	4	0	17	15	0	0	75
Tehran (Tehran)	23	30	3	2	37	33	8	0	136
West Azerbaijan (Urmia)	13	11	0	0	16	9	0	0	49
Yazd (Yazd)	27	29	0	0	11	6	2	0	75
Subtotal	272	244	20	8	228	169	24	0	965
Total	544				421				

F: female, M: male, N: nymph, E: egg.

Two cockroach species (*P*. *americana* and *B*. *germanica*) were collected and stored in 50ml centrifuge tubes and delivered to the laboratory of insect molecular biology, School of Public Health, Tehran University of Medical Sciences. The cockroaches were identified using relevant taxonomic keys and descriptions [39]. A subset of individuals from each species and location was selected and preserved at –80°C for subsequent molecular investigation.

### Tissue dissection

The legs and wings of specimens were removed before isolating the gut and reproductive tissues. Tissue-specific dissection was carried out on each sample and dissected tissues, including the reproductive organs and alimentary canal, of each specimen were individually placed into small Petri dish in diameter size of 35 mm on ice to prevent DNA degradation. All dissection equipment and microscope slides were thoroughly wiped with 70% ethanol before commencing dissection of each sample.

### DNA extraction and PCR amplification

DNA was extracted from each specimen using a QIAamp DNA mini kit (Qiagen, Hilden, Germany) according to the manufacturer’s protocol, and the extracted DNA was stored at minus 20°C. In this study, two molecular markers, *Wolbachia* surface protein (*wsp*) and the citrate synthase (*gltA*) genes, were used to detect *Wolbachia* infection. The *wsp* general primers were 81F (5′-TGGTCCAATAAGTGATGAAGAAAC-3′) and 691R (5′-AAAAATTAAACGCTACTCCA-3′) [40] and the *gltA* primers, were WgltAF1 (5′-TACGATCCAGGGTTTGTTTCTAC-3′) and WgltARev2 (5′-CATTTCATACCACTGGGCAA-3′) [41] and these served to confirm *Wolbachia* identity.

For the *wsp* gene amplification, extracted DNA samples from the dissected tissues were screened by PCR in a thermocycler (Eppendorf, Hamburg, Germany) using the following protocol: initial denaturation at 95°C for 4 min; 35 cycles of 94°C 1min, 55°C 1min and 72°C 1min and final elongation step of 72°C for 10 min. For the *gltA* gene amplification, initial denaturation at 95°C for 2 min; two cycles of denaturation at 95°C for 2 min, annealing at 60°C for 1 min and extension at 72°C for 1 min; 35 cycles of denaturation at 95°C for 30s, annealing at 60°C for 1 min and extension at 72°C for 45s; final extension at 72°C for 10 min.

All PCR procedures were performed in reaction mixtures consisting of 12.5μl of *Taq* DNA Polymerase Master Mix RED (Denmark), 3μl of extracted DNA, and 1μl each of 5μM forward and reverse primers for *Wolbachia* PCR screens. Double-distilled water was used to top up the reaction mixture to a final volume of 25μl. PCR amplification of positive and negative controls was also conducted simultaneously. The negative controls were prepared with ddH_2_O and DNA from a male *Anopheles stephensi* mosquito; a positive control was prepared in our laboratory using DNA extracted from *Drosophila melanogaster* which harbours *Wolbachia wMel*Pop strain. Amplicons were separated by gel electrophoresis on 1.5% agarose gel stained with green viewer (Parstous, Iran) and visualised under an ultraviolet transilluminator.

### Gene sequencing and phylogenetic analysis

**Fig 2:**
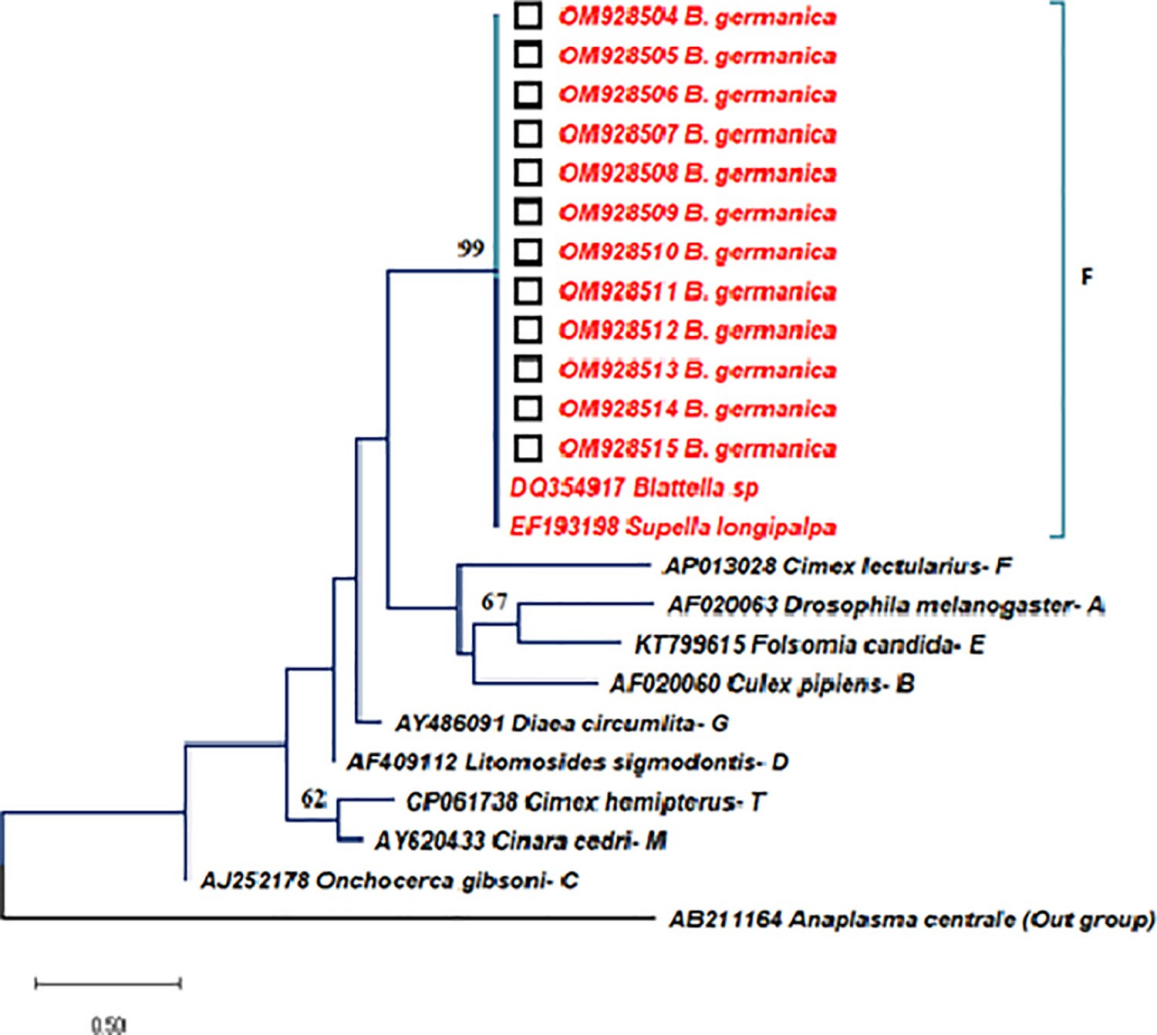
**Phylogenetic relationship inferred from 443 bp of *wsp* gene sequences of *B*. *germanica* from Iran (OM928504-OM928515 that are shown by black square) and some representative sequences of *Wolbachia* strains belonging to supergroups A, B, C, D, E, F, G, and M obtained from Genbank database.** The Genbank ID numbers, the host species, and the *Wolbachia* supergroup are shown in the tree branches respectively. The phylogenetic tree was created using Neighbour Joining–Kimura. Scale bar shows genetic distance. Numbers in the tree represent the bootstrap value (bootstrap values below 50% are not shown at the nodes).

**Fig 3:**
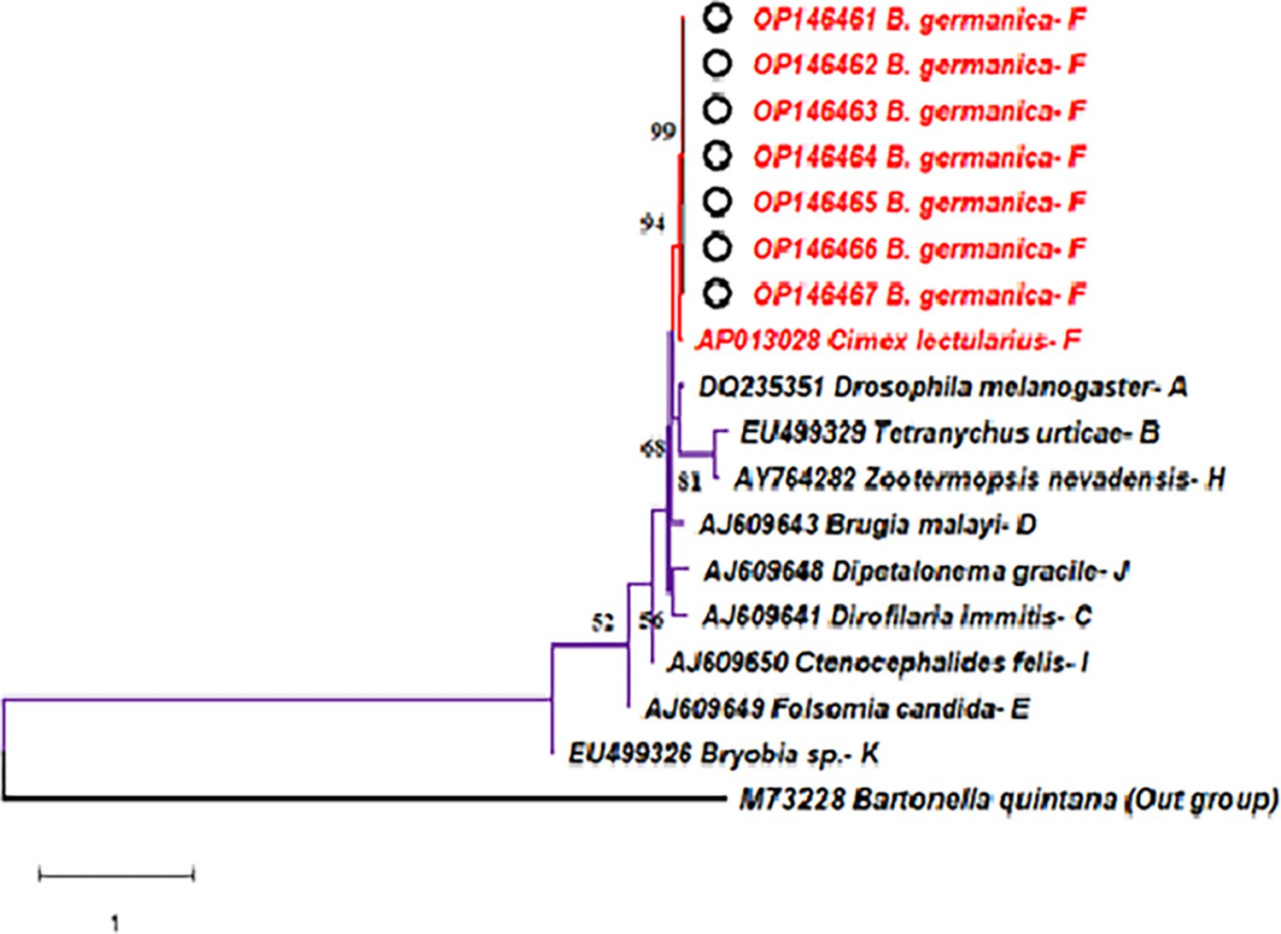
**Phylogenetic relationship inferred from 599 bp of *gltA* gene sequences of *B*. *germanica* from Iran (OP146461-OP146466 that are shown by black circles) and some representative sequences of *Wolbachia* strains belonged to supergroups A, B, C, D, E, F, H, I, J, and K obtained from Genbank database.** The Genbank ID numbers, the host species, and the *Wolbachia* supergroup are shown in the tree branches respectively. The phylogenetic tree was created using Neighbour Joining–Kimura. Scale bar shows genetic distance. Numbers in the tree represent the bootstrap value (bootstrap values below 50% are not shown at the nodes).

The criteria set to confirm *Wolbachia* infection were based on successful amplification of the molecular markers. Furthermore, samples that met this criterion were sequenced by bidirectional sequencing at Genomin Sequencing Centre, Iran. A subset of the PCR products of *wsp* and *gltA* gene representatives of different locations were purified from gels using a gel purification kit and subjected to sequencing. Sequencing was performed using an ABI 3730 sequencer machine. The resultant sequences were checked to correct ambiguities. Homologies with the available sequence data in GenBank were checked by using basic local alignment search tool (BLAST) analysis software (**www.ncbi.nlm.nih.gov/BLAST**). A subset of consensus sequences for both loci was deposited in the GenBank database (Table 2). A subset of the available representative *Wolbachia wsp* and *gltA* sequences of nine supergroups (A-G, M, and T for wsp gene and A-F, and H-K for gltA gene) were acquired from public databases (**http://www.ncbi.nlm**) for phylogenetic analysis (Table 3). Owing to the different lengths of these sequences, all those used for alignment were trimmed to obtain consistent regions that were 443 bp for *wsp* and 599 bp for *gltA* genes, respectively. Multiple alignments of the sequences were carried out using the Clustal W algorithm in software MEGA X [42]. The short sequence reads were excluded. Pairwise sequence divergence, using Kimura’s 2-parameter distance algorithm, and neighbour joining (NJ) tree, shown in Figs 2 and 3, were processed in MEGA X. The robustness of all phylogenetic trees was tested with a bootstrapping value in NJ. The *wsp* sequence of *Anaplasma centrale* (Genbank ID: AB211162) and *gltA* sequence of *Bartonella quintana* (Genbank ID number: M73228) were acquired from Genbank and used as outgroups for each gene (Table 2).

**Table 2:** Representative *wsp* and *gltA Wolbachia* type sequences used in this study.

Molecular marker	Host scientific name	Host common name	*Wolbachia* super group	GenBank ID	References
***wsp***	*Drosophila melanogaster*	Fruit fly	A	AF020072	[40]
	*Culex pipiens*	House mosquito	B	KM401551	[43]
	*Onchocerca gibsoni*	Nematodes	C	AJ252178	[44]
	*Litomosides sigmodontis*	Filarial worm	D	AF409112	[45]
	*Folsomia candida*	Springtail	E	KT799615	[46]
	*Cimex lectularius*	Bed bug	F	AP013028	[47]
	Blattella sp.	Cockroach	F	DQ354917	[38]
	*Supella longipalpa*	Brown banded cockroach	F	EF193198	
	*Blattella germanica*	German cockroach	F	OM928504-OM928515	This study
	*Diaea circumlita*	NA	G	AY486091	[48]
	*Cinara cedri*	Cedar bark aphid	M	AY620433	[49]
	*Cimex hemipterus*	Bed bug	T	CP061738	[50]
	*Anaplasma centrale* (Outgroup)	NA	NA	AB211164	[51]
***gltA***	*Drosophila melanogaster*	Fruit fly	A	DQ235351	[52]
	*Tetranychus urticae*	Red spider mite	B	EU499329	[53]
	*Dirofilaria immitis*	Dog heart worm	C	AJ609641	[41]
	*Brugia malayi*	Roundworm	D	AJ609643	
	*Folsomia candida*	Springtail	E	AJ609649	
	*Cimex lectularius*	Bed bug	F	AP013028	[47]
	*Blattella germanica*	German cockroach	F	OP146461-OP146467	This study
	*Zootermopsis nevadensis*	Nevada termite	H	AY764282	[54]
	*Ctenocephalides felis*	Cat flea	I	AJ609650	[41]
	*Dipetalonema gracile*	NA	J	AJ609648	
	Bryobia sp.	Brown mites	K	EU499326	[53]
	*Bartonella quintana* (Outgroup)	NA	NA	M73228	[55]

**Table 3:** Prevalence of *Wolbachia* infection in cockroaches collected from different localities of Iran.

No	Location	Coordinate	Species	No. of infected/Total (%)	Positive samples			
					Adult		Immature	
					F	M	N	E
1	Ardabil	38°22’59.4”N 47°40’09.9”E	*B*.* germanica*	4/21(19.04)	3	1	0	0
			*P*.* americana*	0/14	0	0	0	0
2	Fars	29°35’46.8”N 52°28’58.1”E	*B*.* germanica*	12/65(18.46)	8	4	0	0
			*P*.* americana*	0/20	0	0	0	0
3	Ilam	33°37’50.3”N 46°23’14.6”E	*P*.* americana*	0/61	0	0	0	0
4	Isfahan	32°29’36.0”N 51°46’18.1”E	*B*.* germanica*	10/47(21.27)	6	4	0	0
			*P*.* americana*	0/21	0	0	0	0
5	Kerman	30°12’49.2”N 57°01’37.3”E	*B*.* germanica*	11/48 (22.91)	9	1	1	0
			*P*.* americana*	0/31	0	0	0	0
6	Razavi khorasan	36°20’00.6”N 59°30’43.8”E	*B*.* germanica*	10/39 (25.64)	8	2	0	0
			*P*.* americana*	0/42	0	0	0	0
7	Khuzestan	32°22’02.1”N 48°24’25.4”E	*B*.* germanica*	9/49(18.36)	4	5	0	0
			*P*.* americana*	0/33(0)	0	0	0	0
8	Kohgiluyeh&Boyerahmad	30°51’45.3”N 51°27’37.2”E	*B*.* germanica*	8/40(20)	5	2	0	1
			*P*.* americana*	0/12	0	0	0	0
9	Mazandaran	36°35’05.5”N 52°59’25.4”E	*B*.* germanica*	7/45(15.55)	5	2	0	0
			*P*.* americana*	0/32	0	0	0	0
10	Sistan & Baluchestan	29°29’43.1”N 60°48’05.9”E	*B*.* germanica*	14/53(26.41)	10	2	2	0
			*P*.* americana*	0/32	0	0	0	0
11	Tehran	35°43’27.4”N 51°18’22.0”E	*B*.* germanica*	9/58(15.51)	5	3	0	1
			*P*.* americana*	0/78	0	0	0	0
12	West Azerbaijan	37°36’55.9”N 45°03’24.9”E	*B*.* germanica*	6/24(25)	5	2	0	0
			*P*.* americana*	0/25	0	0	0	0
13	Yazd	31°34’49.4”N 54°25’31.1”E	*B*.* germanica*	11/56(19.64)	4	7	0	0
			*P*.* americana*	0/19	0	0	0	0
**Total**			*B*.* germanica*	112/544 (20.6)	72	35	3	2
			*P*.* americana*	0/421(0)	0	0	0	0

(F: female, M: male, N: nymph, E: egg)

### Data analysis

Statistical significance was determined as P < 0.05. All statistical analyses were performed in SPSS statistics version 21.

## Results

### *Wolbachia* detection and prevalence

A total of 965 cockroaches, representing two species, were collected from 13 localities in Iran (Fig 1). PCR assays, using the primers described in Materials and Methods, gave the expected amplification products of 632 bp and 659 bp, for *wsp* and *gltA* respectively. Overall, only *B*. *germanica* was found to host *Wolbachia*. The other cockroach species (*P*. *americana*) showed no PCR amplification products. From a total of 544 German cockroaches screened by *wsp* marker, 95 specimens (17.46%) were found to be infected with *Wolbachia* (Table 3). The *wsp*-negative samples were re-evaluated with the *gltA* gene primers, and 17 out of 449 *wsp*-negative samples were found to be *gltA*-positive; thus, the totals number of *Wolbachia*-infected German cockroaches was 112 out of 544 (20.6%). The prevalence of *Wolbachia* infection was almost twice as high in females as in males (59 versus 32), which is statistically significant (P<0.001).

The Blast search of the *wsp* and *gltA* sequences of infected *B*. *germanica* revealed a high homology, with the F supergroup of *Wolbachia* strains found in *Cimex lectularius* (AP013028), *Blattella sp* (DQ354917), and *Supella longipalpa* (EF193198) (Figs 2 and 3). Following Vaishampayan et al’s finding, the *wsp* sequences obtained in this study can be classified into *Wolbachia* F supergroup [36]. According to our knowledge, this is the first report of *Wolbachia* infection in *B*. *germanica* in Iran (Table 3).

The sequences generated during this study have been deposited in the Gen Bank database (OM928504-OM928515 for *wsp* and OP146461-OP146467 for *gltA* sequences).

### Phylogenetic analysis

The neighbour joining phylogenetic tree between the *Wolbachia* strain identified in this study and other known available *Wolbachia* strains are shown in Figs 2 and 3. Fig 2 shows a phylogenetic tree inferred from 433 bp of *wsp* gene sequences from Iranian isolates of *B*. *germanica* and some representative sequences of *Wolbachia* strains belonging to supergroups A, B, C, D, E, F, G, and M obtained from Genbank database. The phylogenetic analysis showed that *Wolbachia* strains identified from the Iranian specimens were closely associated and clustered with the *Wolbachia* strains presented in *Blattella* sp (DQ354917) and *Supella longipalpa* (EF193198) of supergroup F (Fig 2). In addition, in the phylogenetic analysis of *gltA* sequences, independent of the method for tree reconstruction, the cockroach *Wolbachia* sequences from the Iranian German cockroaches clustered with supergroup F (Fig 3) and led to similar tree topologies as found with the *wsp* gene. Phylogenetic analysis of the *Wolbachia* sequences with Maximum likelihood (ML), Neighbour Joining (NJ) and Maximum Parsimony (MP) methods showed almost similar topology.

## Discussion

In the current research we found that only about twenty percent of *B*. *germanica* specimens collected from the 13 provinces of Iran were positive for *Wolbachia*. This result concurs with the study of Vaishampayan et al in India, which reported that 20% of *B*. *germanica* examined harboured *Wolbachia* [38]. Also, we found no *Wolbachia* infection in American cockroaches. The low rate of *Wolbachia* infection in the German cockroaches and the lack of infection in the American cockroaches may indicate a lack of dependence, or a very low dependence, of the cockroach species on the endosymbiont for their survival and reproduction, in what is known as obligatory relationships [27, 56, 57]. Therefore, the low level of *Wolbachia* infection in cockroaches negates the possibility of *Wolbachia* being an obligatory endosymbiont in these cockroach species. Also, it is possibility that *Wolbachia* may have some negative effects on the fitness of cockroaches, which would counteract the possibility of *Wolbachia* expanding in the population through providing fitness advantages.

The low rate or lack of *Wolbachia* infection in the cockroaches may change in future because it is shown that changing the gut microbiota composition with antibiotic treatment enhanced *Wolbachia* density in *Drosophila melanogaster* [58]. We have no evidence to assess the impact of antibiotic treatment on the incidence and frequency of *Wolbachia* in German cockroaches, however, cockroaches are exposed to antibiotics in places such hospitals [59, 60] and most bacterial agents isolated from cockroaches are antidrug-resistant and antibiotic-resistant [61]. These situations provide cockroaches with diverse antibiotic treatments which may result in raising *Wolbachia* density in future and could be the subject of future studies.

The *Wolbachia* strain found in German cockroaches in this study belongs to supergroup F, which is consistent with previous studies indicating supergroup F in other cockroaches, such as *Supella longipalpa* and *Blattella* sp [38]. *Wolbachia* supergroup F has also been detected in bedbugs and nematodes. The *Wolbachia* supergroup F is essential for the bedbugs’ growth and reproduction because the bacterium provides B vitamins, which are deficient in their blood-based diet [27]. Therefore, the *Wolbachia* strain might promote persistence by providing fitness advantages to the German cockroach via nutrient supplementation [27, 62]. However, we do not yet know if this strain could provide any nutrient supplement for German cockroaches, and this needs to be determined by further studies.

*Wolbachia* strains are divided into 20 supergroups, ranging from A to U (G was not considered anymore) which diverged around 100 million years ago, first in filarial nematodes and then infecting arthropods [63]. In this study we found F supergroup in the cockroaches which is also found in distantly related host species including nematodes and domestic indoor pests (*Cimex*, *Supella* and *Blattella*). *Wolbachia* is transmitted either vertically between host generations or horizontally to other individuals and species through a mechanism called host shift (HS) [63–65].

German or American cockroaches are omnivorous synanthropic insects, frequently encountering high loads of diverse microbes, and are reservoirs and vectors of several pathogens particularly pathogenic bacteria [61, 66–68]. For example, Dokor showed that about a quarter of the microorganisms isolated from cockroaches are food-borne pathogens including *Escherichia coli* O157:H7, *Staphylococcus aureus*, *Bacillus cereus*, *Shigella dysenteriae*, *Salmonella enterica* subsp. *enterica* serovar Typhi, *Rotavirus*, *Aspergillus fumigatus*, and *Cryptosporidium parvum* [66]. Although *Wolbachia* has been shown to protect insects from a range of microbial and eukaryotic pathogens including viruses, *Plasmodium* and filarial nematodes [31–37], there is no strong evidence that *Wolbachia*-infected insects can be protected against pathogenic bacteria. In an experiment, no difference in mortality was observed in the *Drosophila simulans* lines with five different *Wolbachia* strains or without *Wolbachia* when the lines were challenged with the pathogenic bacteria. Similarly, no antibacterial protection or upregulation of the antibacterial immune genes was observed for *D*. *melanogaster* infected with *Wolbachia* compared to paired flies without *Wolbachia*. It was suggested that *Wolbachia*-mediated antibacterial protection is not universal in insects and furthermore that the mechanisms of antibacterial and antiviral protection are independent [69]. In another study it was found that *D*. *melanogaster* flies harbouring no endosymbionts, those carrying both *Spiroplasma* and *Wolbachia*, and those containing *Wolbachia* only had parallel survival rates following infection with the virulent insect pathogen *Photorhabdus luminescens* and non-pathogenic *Escherichia coli* bacteria [70]. Also, *Wolbachia* presence did not provide a protective advantage against entomopathogenic fungi, *Beauveria bassiana* and *B*. *brongniartii*, in two important mosquito vectors, *Aedes albopictus* and *Culex pipiens* that naturally carry *Wolbachia* [71]. Taken together these results plus high microbial loads, we suggest that presence of *Wolbachia* supergroup F may not provide protection in the German cockroach species. Also, having found *Wolbachia* supergroup F in German cockroaches warrants further studies to determine if *Wolbachia* supergroup F can manipulate the cockroach’s reproduction system through such means as cytoplasmic incompatibility (CI), induction of parthenogenesis (IP), male-killing (MK), or feminization of genetic males (MF) [46, 72–74].

In this study the cockroaches were molecularly screened for *Wolbachia* DNA using two primer sets targeting partial *wsp* and *gltA* genes. Comparing the results of this study pointed the discrepancy in results between the primer pairs where *Wolbachia* DNA detected at the *wsp* locus was less than at the *gltA* locus. Baldo et al [75] showed that *wsp* gene has a mosaic structure with four hypervariable regions (HVRs), which provide a reasonable explanation for the negative results. It is possible that there is a trade-off between sensitivity and specificity of primer sets and certain primer sets can be more efficient than others, but that no single protocol can ensure the specific detection of all known *Wolbachia* infections [76].

In the present study we found that there was a sex bias toward infection in females of *B*. *germanica*, with *Wolbachia* prevalence in females being higher than in males. *Wolbachia* infections tend to confer reproductive advantages on their female hosts, which is the sex responsible for vertical transmission of the bacteria from one generation to another, leading to increased prevalence and propagation within the host populations [74]. Higher *Wolbachia* infection in females has already been reported in few hosts, such as hard ticks [77], fruit flies [78], and fleas [79]. Low levels of *Wolbachia* infection in male cockroaches also tends to support rejection of the possibility of *Wolbachia* as an obligatory endosymbiont, at least in males. Finally, the low occurrence of *Wolbachia* in male cockroaches may suggests the absence of robust CI in German cockroaches.

## Conclusion

In conclusion, we found low or no *Wolbachia* infection in German and American cockroaches, respectively, calling for additional surveys of hidden fitness, nutrition, or protection properties, the reproductive manipulation such as CI occurrence, and underlying systems with sex-bias differences in *Wolbachia* persistence. Nevertheless, the long evolutionary history of *Wolbachia*’s interaction with invertebrate hosts and its adaptations for germ line transmission contribute to the value of *Wolbachia* for control of insect pests.
